# Effects of Ratoon Rice Cropping Patterns on Greenhouse Gas Emissions and Yield in Double-Season Rice Regions

**DOI:** 10.3390/plants13111527

**Published:** 2024-05-31

**Authors:** Jinbiao Xiang, Liusheng Zhong, Zhixiong Yuan, Liqin Liang, Zhangzhen Yang, Yanmei Xiao, Zhiqiang Fu, Pan Long, Cheng Huang, Ying Xu

**Affiliations:** 1 College of Agronomy, Hunan Agricultural University, Changsha 410128, China; 2 Key Laboratory of the Ministry of Education for Crop Physiology and Molecular Biology, Hunan Agricultural University, Changsha 410128, China

**Keywords:** cropping pattern, ratoon rice, greenhouse gas, global warming potential

## Abstract

The ratoon rice cropping pattern is an alternative to the double-season rice cropping pattern in central China due to its comparable annual yield and relatively lower cost and labor requirements. However, the impact of the ratoon rice cropping pattern on greenhouse gas (GHG) emissions and yields in the double-season rice region requires further investigation. Here, we compared two cropping patterns, fallow-double season rice (DR) and fallow-ratoon rice (RR), by using two early-season rice varieties (ZJZ17, LY287) and two late-season rice varieties (WY103, TY390) for DR, and two ratoon rice varieties (YLY911, LY6326) for RR. The six varieties constituted four treatments, including DR1 (ZJZ17 + WY103), DR2 (LY287 + TY390), RR1 (YLY911), and RR2 (LY6326). The experimental results showed that conversion from DR to RR cropping pattern significantly altered the GHG emissions, global warming potential (GWP), and GWP per unit yield (yield-scaled GWP). Compared with DR, the RR cropping pattern significantly increased cumulative methane (CH_4_), nitrous oxide (N_2_O), and carbon dioxide (CO_2_) emissions by 65.73%, 30.56%, and 47.13%, respectively, in the first cropping season. Conversely, in the second cropping season, the RR cropping pattern effectively reduced cumulative CH_4_, N_2_O, and CO_2_ emissions by 79.86%, 27.18%, and 30.31%, respectively. RR led to significantly lower annual cumulative CH_4_ emissions, but no significant difference in cumulative annual N_2_O and CO_2_ emissions compared with DR. In total, the RR cropping pattern reduced the annual GWP by 7.38% and the annual yield-scaled GWP by 2.48% when compared to the DR cropping pattern. Rice variety also showed certain effects on the yields and GHG emissions in different RR cropping patterns. Compared with RR1, RR2 significantly increased annual yield while decreasing annual GWP and annual yield-scaled GWP. In conclusion, the LY6326 RR cropping pattern may be a highly promising strategy to simultaneously reduce GWP and maintain high grain yield in double-season rice regions in central China.

## 1. Introduction

Methane (CH_4_), nitrous oxide (N_2_O), and carbon dioxide (CO_2_) are the most important anthropogenic greenhouse gases in the atmosphere [1,2]. Their emissions can cause changes to the global climate and damage to natural ecosystems, leading to significant socio-economic losses [3,4]. Agricultural production is a significant source of anthropogenic greenhouse gas emissions [5,6,7]. It has been estimated that greenhouse gas emissions from agricultural production account for approximately 10.00–12.00% of the global total anthropogenic greenhouse gas emissions [8]. China is the largest rice-producing country in the world, with an average annual rice production of 210 million tons, which accounts for approximately 29.00% of global rice production [9]. Rice paddies are not only a major source of anthropogenic CH_4_ emissions but also a source and sink of N_2_O [10,11]. CH_4_ and N_2_O emissions from rice paddies account for approximately 30.00% and 11.00% of the total global agricultural CH_4_ and N_2_O emissions, respectively [12]. Therefore, it is important to increase rice yield, ensure food security, reduce greenhouse gas emissions from rice paddies, and mitigate global climate change to ensure sustainable rice production in China. Currently, the adoption of appropriate agricultural management practices and rice varieties is a common strategy to reduce greenhouse gas emissions from rice production [13,14]. Therefore, it is of great significance to seek and combine rational, scientific, and sustainable agricultural management practices and rice varieties to better control the global greenhouse effect and increase rice yields.

With changes in socio-economic conditions and increasing demand for food security, the rice paddy cropping patterns in the middle and lower reaches of the Yangtze River, the main rice production region in China, are constantly adjusted [15,16,17]. Compared with that of double-season rice, the cultivation of ratoon rice can effectively shorten the growth period and save labor, seeds, and water, thereby improving the quality and economic benefits of rice [18,19,20,21]. As a result, ratoon rice cultivation has become an important simplified cultivation system, playing a crucial role in ensuring China’s food security in double-season rice planting regions. Due to different field management practices (such as water regime, intensive tillage, and fertilization rate), the conversion from a double-season rice cropping pattern to a ratoon rice cropping pattern will alter the soil physicochemical properties, which will affect the root growth and microorganism activities and further influence organic matter decomposition, thereby resulting in changes in greenhouse gas emissions, as well as yield. Currently, although emissions of CH_4_ and N_2_O from double-season rice and ratoon rice cropping patterns have been documented, they have focused only on the growing season of rice, and little is known about the impact of ratoon rice cropping pattern on greenhouse gas emissions over the whole annual rotation in the double-season rice region [21]. Moreover, field measurements of greenhouse gas emissions and their trade-offs have rarely been taken over a whole annual ratoon rice cropping rotation, which leads to a lack of quantitative information regarding greenhouse gas emissions and their trade-offs during the non-rice season. Therefore, it is necessary to extend previous studies by evaluating the estimate of greenhouse gas emissions and grain yield in the ratoon rice cropping pattern over the entire year in the double-season rice region.

The flux of greenhouse gases varies with rice-based cropping patterns in fields [22,23,24,25]. An effective rice-based cropping pattern strategy to mitigate greenhouse gases requires considering multiple greenhouse gases simultaneously when evaluating the impacts on radiative forcing [24,25]. The global warming potential (GWP) was introduced to estimate the potential future impacts of emissions of different gases on the climate system and radiative properties [26]. Another concept for relating rice-based cropping patterns to the GWP is GWP per unit yield (yield-scaled GWP). This term is calculated by dividing the GWP by crop yield and was introduced to assess the climatic impacts of agriculture per kg of yield [24,27]. Previous studies have demonstrated that the ratoon rice cropping pattern generally has a lower carbon footprint, GWP, and yield-scaled GWP compared with the double-season rice cropping pattern [21,23,28,29]. However, in most previous studies, few studies have simultaneously examined the emission dynamics of these three greenhouse gases (CH_4_, N_2_O, and CO_2_) and their GWP under ratoon rice cropping patterns in the double-season rice region on a field scale. Therefore, to have a more accurate and scientific assessment of the GWP and yield-scaled GWP, further information about the annual GWP and annual yield-scaled GWP under ratoon rice cropping patterns in the double-season rice region is necessary.

Our objectives were to (1) monitor the emission patterns of CH_4_, N_2_O, and CO_2_ and grain yield under double-season rice and ratoon rice cropping patterns in Hunan Province, a main planting region of double-season rice with ample solar radiation and heat resources; (2) assess the combined effects of the GWP and yield-scaled GWP created by double-season rice and ratoon rice cropping patterns; and (3) optimize ratoon rice cropping patterns combined with high-quality rice varieties without compromising grain yield in central China.

## 2. Results

### 2.1. Crop Grain Yield

**Figure 1:**
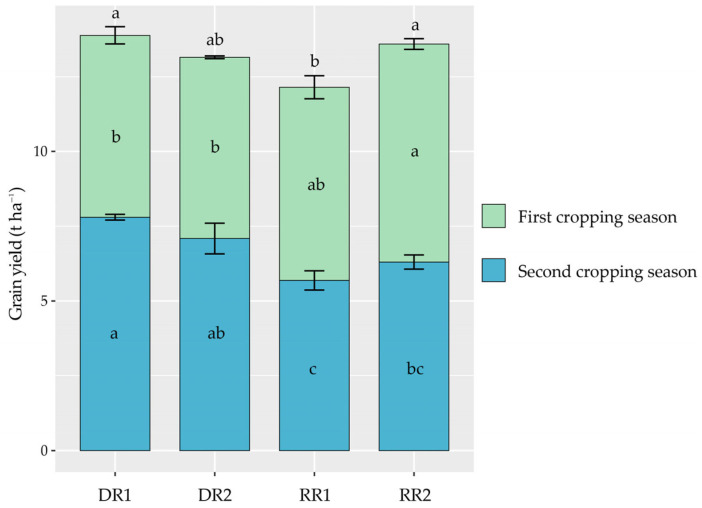
Crop grain yields during crop growing seasons under different cropping patterns. DR, fallow-double season rice; RR, fallow-ratoon rice. Early-season varieties Zhongjiazao 17 (ZJZ17) and Liangyou 287 (LY287); late-season varieties Wuyou 103 (WY103) and Taiyou 390 (TY390); ratoon rice varieties of Y Liangyou 911 (YLY911) and Liangyou 6326 (LY6326). DR1, ZJZ17 + WY103; DR2, LY287 + TY390; RR1, YLY911; and RR2, LY6326. The vertical bars above the columns represent standard errors (*n* = 3). Different lowercase letters indicate significant differences in the same season (annual) across cropping patterns at the *p* < 0.05 level.

Grain yield was significantly affected by the cropping season (Table 1, Figure 1). The grain yield of the two varieties in the early rice season was significantly lower than that in the main crop season of Liangyou 6326 (LY6326) (Figure 1). On the contrary, compared with the average yield in the late rice season, the grain yield of Y Liangyou 911 (YLY911) and LY6326 in the ratoon crop season decreased by 23.66% and 15.35%, respectively (Figure 1). In terms of annual crop yield, compared with the DR1 (Zhongjiazao 17 (ZJZ17) + Wuyou 103 (WY103)) cropping pattern, the DR2 (Liangyou 287 (LY287) + Taiyou 390 (TY390)), RR1 (YLY911) and RR2 (LY6326) cropping patterns had decreases in grain yield by 5.30%, 12.50% and 2.09%, respectively (Figure 1).

### 2.2. CH_4_ Emission

**Figure 2:**
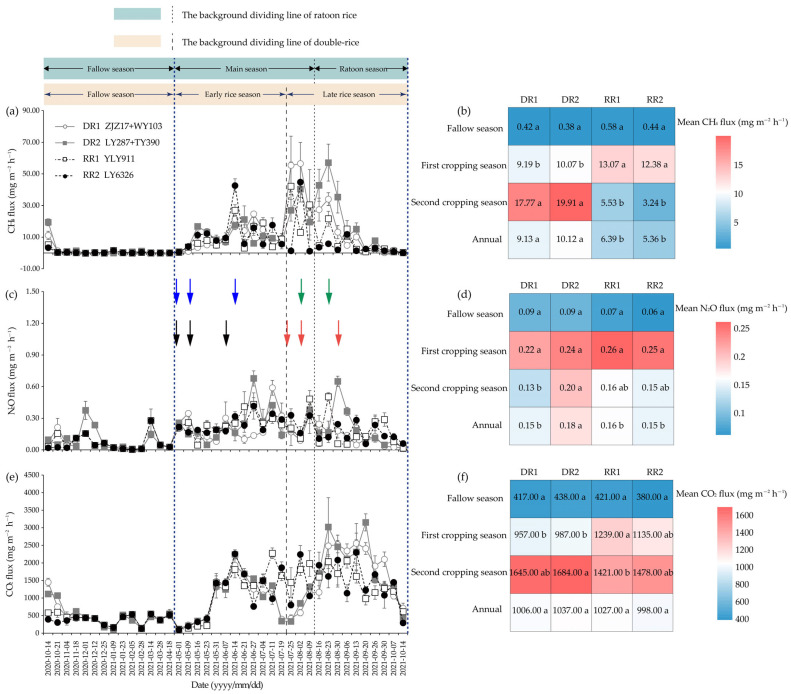
Seasonal variation and mean flux emissions of CH_4_ (**a**,**b**), N_2_O (**c**,**d**), and CO_2_ (**e**,**f**) under different cropping patterns. DR, fallow-double season rice; RR, fallow-ratoon rice. Early-season varieties Zhongjiazao 17 (ZJZ17) and Liangyou 287 (LY287); late-season varieties Wuyou 103 (WY103) and Taiyou 390 (TY390); ratoon rice varieties of Y Liangyou 911 (YLY911) and Liangyou 6326 (LY6326). DR1, ZJZ17+ WY103; DR2, LY287 + TY390; RR1, YLY911; and RR2, LY6326. The data are presented as mean (±standard errors). Different lowercase letters indicate significant differences across cropping patterns at the *p* < 0.05 level. Black and red arrows represent the fertilization timing for early rice and late rice, respectively, while blue and green arrows represent the fertilization timing for the main season and ratoon season, respectively.

**Figure 3:**
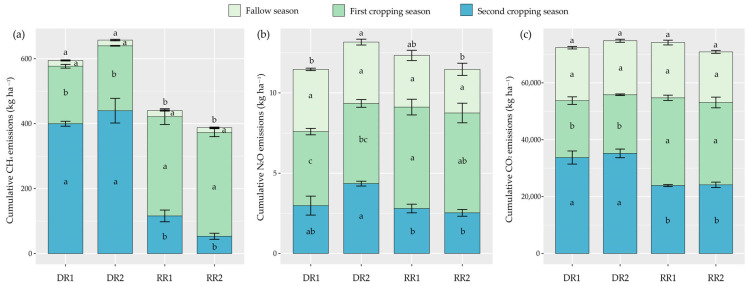
The cumulative emissions of CH_4_ (**a**), N_2_O (**b**), and CO_2_ (**c**) under different cropping patterns. DR, fallow-double season rice; RR, fallow-ratoon rice. Early-season varieties Zhongjiazao 17 (ZJZ17) and Liangyou 287 (LY287); late-season varieties Wuyou 103 (WY103) and Taiyou 390 (TY390); ratoon rice varieties of Y Liangyou 911 (YLY911) and Liangyou 6326 (LY6326). DR1, ZJZ17 + WY103; DR2, LY287 + TY390; RR1, YLY911; and RR2, LY6326. The vertical bars above the columns represent standard errors (*n* = 3). Different lowercase letters indicate significant differences in the same season (annual) across cropping patterns at the *p* < 0.05 level.

The data indicated that CH_4_ fluxes mainly occur during the rice season while approaching zero during the fallow season (Figure 2a). Cropping season, cropping pattern, and their interactions showed significant effects on CH_4_ emissions, while no obvious differences were observed between varieties in the same season of a certain cropping pattern (Table 1, Figure 2b and Figure 3a). In the fallow season, all cropping patterns had low CH_4_ emissions (Figure 2b and Figure 3a). In the first cropping season, the CH_4_ cumulative emissions of LY287, YLY911, and LY6326 were 12.82%, 72.55%, and 80.16% higher than those of ZJZ17, respectively (Figure 3a). In the second cropping season, the CH_4_ cumulative emissions of the two varieties in the ratoon crop season were significantly lower than those in the late rice season (Figure 3a). As a result, the annual (including the fallow, first cropping, and second cropping season) cumulative CH_4_ emissions from DR1, DR2, RR1 and RR2 were 594.58 ± 14.61, 657.34 ± 38.32, 440.52 ± 18.22 and 387.62 ± 10.99 kg CH_4_ ha^−1^, respectively, and there were significant differences between fallow-double season rice (DR) and fallow-ratoon rice (RR) cropping patterns (Figure 3a).

### 2.3. N_2_O Emission

Considerable N_2_O emissions were detected in the rice seasons, particularly right after fertilizer application (Figure 2c). Cropping season had significant effects on N_2_O emissions, while no obvious differences were found between varieties for the same crop season of the same cropping pattern as well (Table 1, Figure 2d and Figure 3b). In the fallow season, all cropping patterns had relatively stable N_2_O emissions (Figure 2d and Figure 3b). In the first cropping season, LY287, YLY911, and LY6326 had 8.35%, 37.05%, and 34.97% higher cumulative N_2_O emissions than ZJZ17, respectively (Figure 3b). However, in the second cropping season, WY103, YLY911, and LY6326 had 31.47%, 35.46%, and 41.81% lower cumulative N_2_O emissions relative to TY390, respectively (Figure 3b). The annual cumulative N_2_O emissions from DR1, DR2, RR1, and RR2 were 11.47 ± 0.57, 13.16 ± 0.29, 12.34 ± 0.56 and 11.47 ± 0.64 kg N_2_O ha^−1^, respectively (Figure 3b).

### 2.4. CO_2_ Emission

Similar CO_2_ flux patterns were observed for different varieties in the same cropping season (Figure 2e). Cropping season had significant effects on CO_2_ emissions (Table 1, Figure 2f and Figure 3c). Cumulative CO_2_ emissions exhibited a similar trend to cumulative N_2_O emissions in various crop seasons and cropping patterns (Figure 3c). In the fallow season, the cumulative CO_2_ emissions accounted for 25.71%, 25.38%, 26.19%, and 25.10% of the annual total CO_2_ emissions from the DR1, DR2, RR1 and RR2 cropping patterns, respectively (Figure 3c). In the first cropping season, the average cumulative CO_2_ emissions of the two varieties under the main crop season were significantly increased by 47.13% compared with those in the early rice season (Figure 3c). Inversely, in the second cropping season, the average cumulative CO_2_ emissions in the late rice season were 1.43-fold that in the ratoon crop season (Figure 3c). The annual cumulative CO_2_ emissions from DR1, DR2, RR1, and RR2 were 72,354.68 ± 2184.08, 74,806.39 ± 1498.93, 74,161.31 ± 2036.38, and 70,882.32 ± 2618.62 kg CO_2_ ha^−1^, respectively (Figure 3c).

### 2.5. The GWP and Yield-Scaled GWP

**Figure 4:**
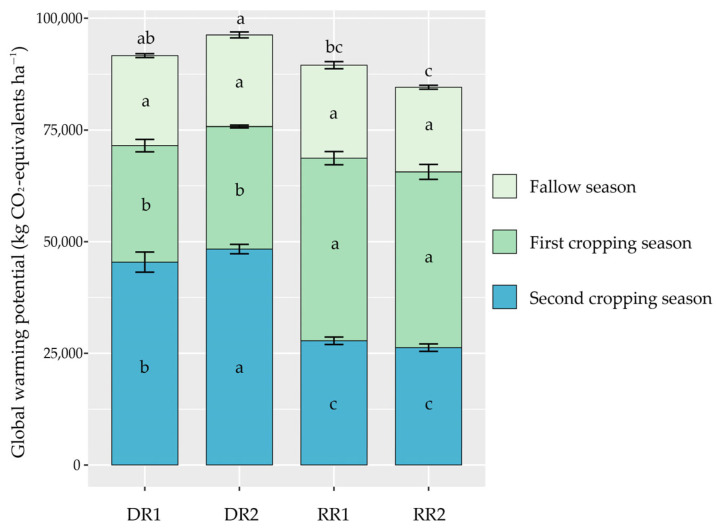
The GWP under different cropping patterns. DR, fallow-double season rice; RR, fallow-ratoon rice. Early-season varieties Zhongjiazao 17 (ZJZ17) and Liangyou 287 (LY287); late-season varieties Wuyou 103 (WY103) and Taiyou 390 (TY390); ratoon rice varieties of Y Liangyou 911 (YLY911) and Liangyou 6326 (LY6326). DR1, ZJZ17 + WY103; DR2, LY287 + TY390; RR1, YLY911; and RR2, LY6326. The vertical bars above the columns represent standard errors (*n* = 3). Different lowercase letters indicate significant differences in the same season (annual) across cropping patterns at the *p* < 0.05 level.

The contribution of CH_4_ (1.79–29.20%) and CO_2_ (68.17–94.91%) emissions varied in different cropping seasons, but N_2_O emissions (1.48–5.58%) always had the least contribution in all seasons (Figure 3 and Figure 4). The cropping season had significant effects on GWP (Table 1, Figure 4). In the fallow season, the GWP did not significantly differ among the cropping patterns (Figure 4). In the first cropping season, the GWP of LY287, YLY911, and LY6326 was 5.10%, 56.68%, and 50.74% higher than that of ZJZ17, respectively (Figure 4). In the second cropping season, the late rice season had a significantly higher (1.73-fold) GWP than the ratoon crop season (Figure 4). In terms of annual GWP, DR1 and DR2 cropping patterns exhibited higher GWP values than RR1 and RR2 cropping patterns, particularly than RR2 cropping pattern (Figure 4). In addition, in all cropping patterns, the GWP in the fallow season accounted for 20.03–23.52%, in the first cropping season for 26.53–47.25%, and in the second cropping season for 29.27–51.90% of the annual total GWP, respectively (Figure 4). These results demonstrated that the RR cropping pattern can reduce the emissions of CO_2_ into the atmosphere in the double-season rice region.

**Figure 5:**
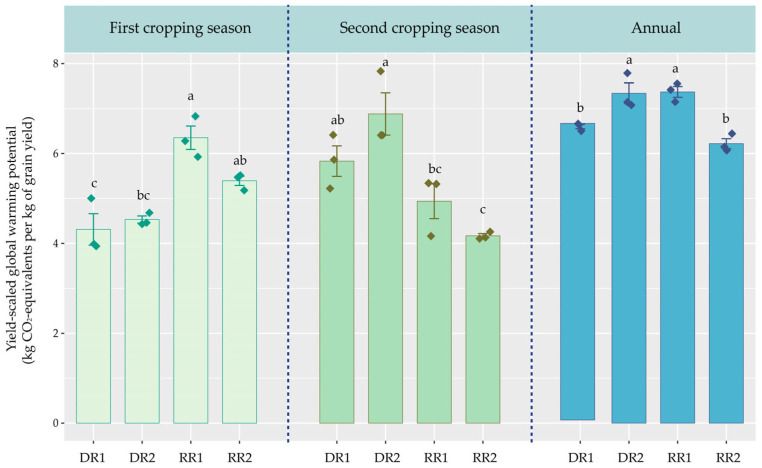
The yield-scaled GWP under different cropping patterns. DR, fallow-double season rice; RR, fallow-ratoon rice. Early-season varieties Zhongjiazao 17 (ZJZ17) and Liangyou 287 (LY287); late-season varieties Wuyou 103 (WY103) and Taiyou 390 (TY390); ratoon rice varieties of Y Liangyou 911 (YLY911) and Liangyou 6326 (LY6326). DR1, ZJZ17 + WY103; DR2, LY287 + TY390; RR1, YLY911; and RR2, LY6326. The vertical bars above the columns represent standard errors (*n* = 3). Different lowercase letters indicate significant differences across cropping patterns at the *p* < 0.05 level.

In the first cropping season, the high GWP of YLY911 led to a higher yield-scaled GWP value (6.35 ± 0.26 kg CO_2_-equivalents per kg of grain yield) compared with the average GWP in the early rice season, while the yield-scaled GWP of ZJZ17 was lower (4.31 ± 0.35 kg CO_2_-equivalents per kg of grain yield) because of a reduction in GWP (Figure 5). In the second cropping season, the yield-scaled GWP in the late rice season was significantly higher than that in the ratoon crop season (Figure 5). In terms of annual yield-scaled GWP, the RR2 cropping pattern had the lowest yield-scaled GWP (6.22 ± 0.11 kg CO_2_-equivalents per kg of grain yield) among all cropping patterns (Figure 5). Compared with the RR2 cropping pattern, the DR1, DR2, and RR1 cropping patterns showed higher annual yield-scaled GWP by 6.10%, 17.96%, and 18.51%, respectively (Figure 5). In summary, cropping patterns and varieties in rice paddy fields should be considered to achieve low-carbon agriculture. 

## 3. Discussion

### 3.1. Impacts of Different Rice Paddy Cropping Patterns on Grain Yield

**Figure 6:**
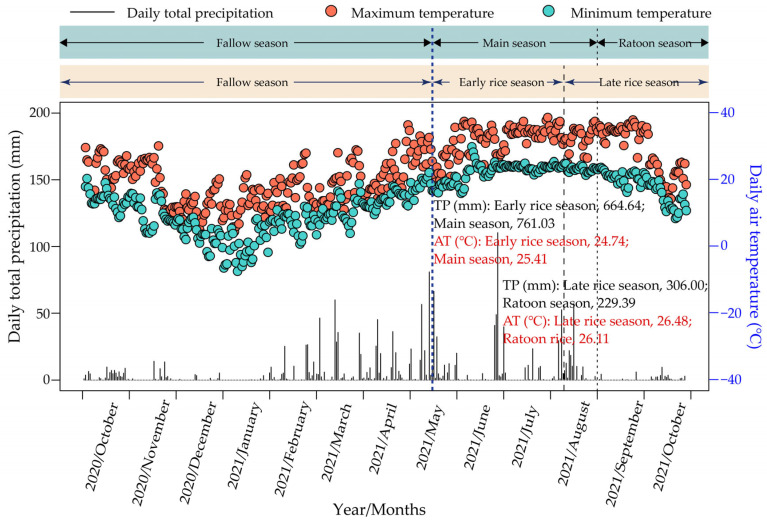
Daily total precipitation, maximum and minimum air temperature during the experimental period in Yanxi, China. TP, total precipitation; AT, average air temperature.

It is commonly believed that an extended growth duration might result in a greater yield [30]. Therefore, the superiority of the main crop in the RR cropping pattern in terms of grain yield over that of the early-season rice in the DR cropping pattern could be ascribed to the longer (by 23 days) growth period of the main crop season compared with that of the early rice season (Figure 1, Table 2). In the second cropping season, the late rice season exhibited a higher grain yield compared to the ratoon crop season (Figure 1). Air temperature significantly affects the yield potential of rice [20,30]. The average daily temperature in the late rice season was higher than that in the ratoon crop season (Figure 6), which might have affected the grain yield potential of the late-season rice. In terms of annual crop yield, our results demonstrated that compared with that of the DR cropping pattern, the annual grain yield of the RR1 cropping pattern was significantly lower, while the RR2 cropping pattern maintained a relatively stable annual grain yield (Figure 1). These results support the view that variety is also a major factor that affects the yield of ratoon rice [18,28].

### 3.2. Impacts of Different Rice Paddy Cropping Patterns on Greenhouse Gas Emissions

**Figure 7:**
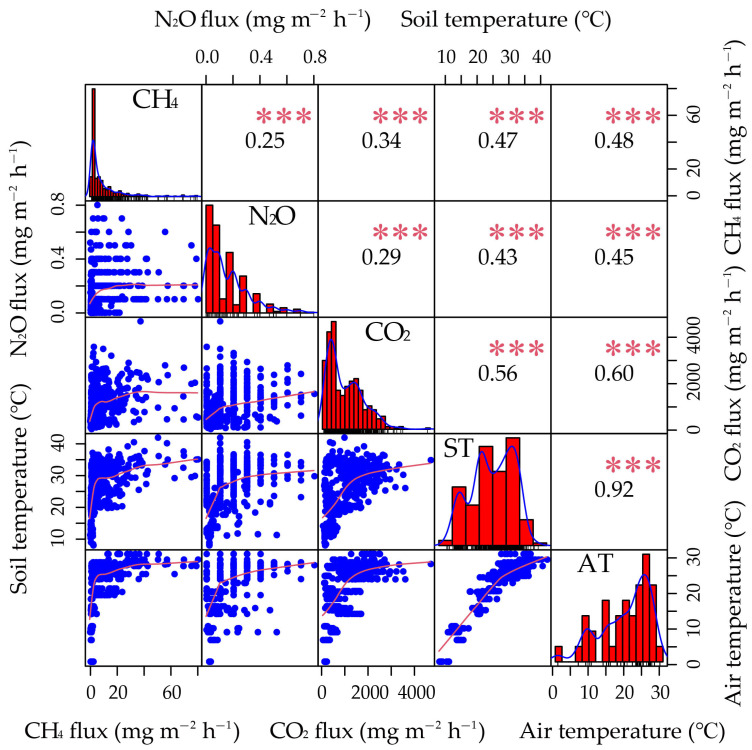
Correlation analysis of greenhouse gas fluxes with soil temperature (ST) and air temperature (AT). The numbers represent the correlation coefficients. *** *p* < 0.001.

Growing evidence has suggested that different cropping patterns affect greenhouse gas emissions by influencing the quantity and quality of carbon and nitrogen (N) inputs from the crops to the soil, the extent of microbial utilization of carbon and N sources in the rhizosphere, and the diversity of carbon and N source metabolism, which will affect the transformation intensity and direction of both inherent and newly input organic matter in the soil, further leading to variations in greenhouse gas production and emission patterns [16,23,25,28,31]. It is possible to select and combine suitable rice varieties and cropping patterns to mitigate greenhouse gas emissions [32]. In this study, the greenhouse gas emissions from paddy fields exhibited distinct seasonal variations (Table 1, Figure 2 and Figure 3), and significant positive correlations were found between greenhouse gas fluxes and the soil and air temperature (Figure 7). These results imply that the temperature of soil and air could, to some degree, explain the seasonal variations in greenhouse gas emissions from rice paddy. It has been reported that higher temperatures can enhance the activity of greenhouse gas-producing bacteria, resulting in higher greenhouse gas emissions [33,34]. Rice plants play a crucial role in greenhouse gas emissions of rice fields, and in previous studies, either a significant difference or no significant difference was observed between different rice varieties in terms of greenhouse gas emissions [5,32]. In this study, no significant differences were found between different rice varieties regarding CH_4_ and CO_2_ emissions in the same season of a certain cropping pattern (Table 1, Figure 2b,f and Figure 3a,c), which could be attributed to the equivalent or counterbalanced CH_4_ and CO_2_ emissions of the two rice varieties. However, a significant difference was found between rice varieties regarding N_2_O emissions (Figure 2d and Figure 3b). Further studies are required to validate and understand the influences of rice varieties on greenhouse gas emissions under diverse cropping patterns, particularly the effects of root-induced alterations in the rhizosphere.

In this study, across all cropping patterns, the contribution of the fallow period to annual CH_4_ emissions ranged from 2.19% to 4.66% (Figure 3a). The soil served as a net source (albeit weakly) rather than a net sink of atmospheric CH_4_ during the fallow season (Figure 3a), which is in line with the findings of a few previous studies of fallow emissions in rice cropping [22]. This phenomenon could be associated with the relatively abundant rainfall and subsequently high soil moisture during the fallow season in the experimental region (Figure 6). Furthermore, the growing season of rice in this study had a dominant contribution to the annual CH_4_ emissions (Figure 2a,b, and Figure 3a), which is consistent with previous reports [11,35]. This study demonstrated that RR cropping patterns significantly reduced CH_4_ emissions relative to DR cropping patterns in the second cropping season (Figure 2b and Figure 3a). This may be attributed to better rice root growth and the subsequent increase in rhizospheric oxygen release in the ratoon crop season under RR cropping patterns, which enhances the activity of CH_4_-oxidizing bacteria and suppresses CH_4_ emissions [32]. Additionally, the water management practices in the ratoon crop season under RR cropping patterns may improve soil aeration, enhance soil oxygen availability, and inhibit the activities of CH_4_-producing bacteria, thereby reducing CH_4_ emissions in rice paddies [5,36].

**Figure 8:**
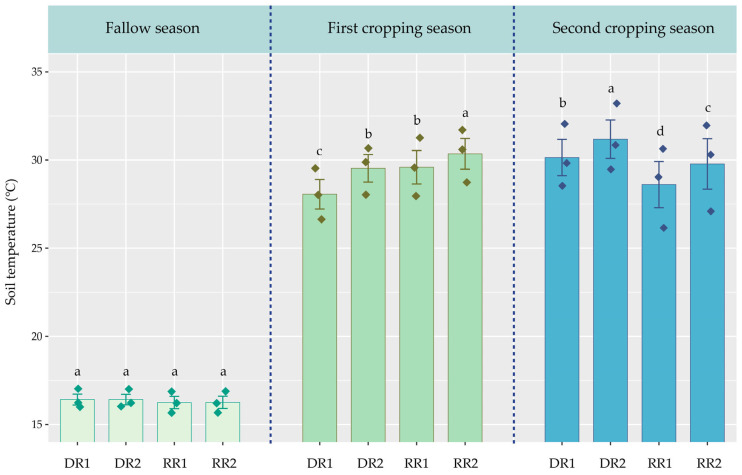
Changes in the soil temperature under different cropping patterns. DR, fallow-double season rice; RR, fallow-ratoon rice. Early-season varieties Zhongjiazao 17 (ZJZ17) and Liangyou 287 (LY287); late-season varieties Wuyou 103 (WY103) and Taiyou 390 (TY390); ratoon rice varieties of Y Liangyou 911 (YLY911) and Liangyou 6326 (LY6326). DR1, ZJZ17 + WY103; DR2, LY287 + TY390; RR1, YLY911; RR2, LY6326. The vertical bars above the columns represent standard errors (*n* = 3). Different lowercase letters indicate significant differences across cropping patterns at the *p* < 0.05 level.

In this study, about 18.16–36.44% of the annual N_2_O emissions were attributed to the winter fallow season (Figure 3b). However, Shang et al. [24] reported that as high as 56.93–93.04% of the annual N_2_O emissions from the double-rice fields in Hunan Province, China were derived from the winter fallow season. The low N_2_O emissions during the fallow season in this study can likely be primarily attributed to the absence of fertilization and the relatively low temperature during that period (Figure 6 and Figure 8). Additionally, our results indicated that RR cropping patterns reduce N_2_O emissions relative to DR cropping patterns in the second cropping season (Figure 2d and Figure 3b). There may be three main reasons for this phenomenon. First, because the cumulative N_2_O emissions under RR cropping patterns may be more sensitive to annual precipitation than those under DR cropping patterns when precipitation infiltrates and carries N fertilizer into the deep soil, it increases the adsorption capacity for ammonium in RR-treated soil, as well as enhances the resistance for N_2_O emissions to the soil surface, thereby reducing N_2_O emissions [37]. Second, the soil temperature under RR cropping patterns is lower than that under DR cropping patterns in the second cropping season (Figure 8), which may slow down the nitrification and denitrification processes, thereby reducing N_2_O emissions [38]. Third, RR cropping patterns may have a higher N utilization efficiency than DR cropping patterns, which can reduce the substrates for soil N_2_O and subsequently reduce N_2_O emissions [39].

In this study, the higher CO_2_ emissions in the main crop and late rice seasons (Figure 2f and Figure 3c) might be partially due to a longer rice growing period than that in the early rice and ratoon crop seasons, respectively (Table 2). In addition, supplementation with “fresh” organic matter could potentially stimulate microbial growth and trigger a positive priming effect to co-metabolize native soil organic matter and fresh carbon, thereby resulting in an increase in CO_2_ emissions [40], which could further explain the higher CO_2_ emissions during the late rice season. Overall, the underlying microbial mechanisms for the aforementioned variations in greenhouse gas emissions still require further exploration in future research.

### 3.3. Impacts of Different Rice Paddy Cropping Patterns on GWP and Yield-Scaled GWP

In this study, the ratoon rice season exhibited significantly lower average GWP (by 42.31%) compared to the late rice season, which might be the primary cause for the lower GWP generated from the RR cropping pattern as opposed to the DR cropping pattern (Figure 4). This finding supports the previous conclusions, suggesting that the ratoon rice system may reduce the carbon footprint relative to the double-season rice system [21,28]. Furthermore, under different cropping patterns, CH_4_ emissions from the rice season had a greater contribution to GWP than N_2_O emissions. Therefore, CH_4_ should be the main target of emission reduction under RR cropping patterns in double-season rice regions, while the warming potential of N_2_O emissions during the fallow season also needs to be considered. There were significant differences in GWP under different cropping patterns, which may be related to factors such as cultivation practices, crop residues, and fertilizer application rates [23,28,41]. In double-season rice regions, RR is a feasible rice cropping pattern to mitigate the greenhouse effect. However, the impacts of RR on soil carbon sequestration, crop carbon sequestration, and overall productivity and economic benefits need to be further investigated.

Yield-scaled GWP is a comprehensive indicator that addresses the dual goals of environmental protection and food security [42]. In this study, differences in yield-scaled GWP were found in different cropping patterns (Figure 5), with the lowest annual value in the RR2 cropping pattern. These results suggest that the yield-scaled GWP in the paddy production system can be reduced by adjusting the cropping system or improving farming management practices. The RR2 cropping pattern in this study could generally maintain an annual grain yield comparable to that in the DR cropping pattern (Figure 1) but resulted in a significantly lower annual GWP (Figure 4), and as a result, the RR2 cropping pattern had a lower yield-scaled GWP than the DR cropping pattern (Figure 5). Thus, the RR2 cropping pattern can be regarded as a feasible system for low-carbon agriculture in the double-season rice regions.

### 3.4. Uncertainty and Limitation

Although the chamber-based technique is the most commonly employed method for measuring CH_4_ and N_2_O in croplands due to its low cost and ease of use [43], the potential impacts of the sampling frequency in both time and space associated with chambers should be taken into account, especially when integrating fluxes to estimate total emissions over extended periods [43]. Ideally, greenhouse gas emissions should be measured frequently enough to capture their peak fluxes. However, in our study, due to the extremely labor-intensive process, heavy workload, and high experimental cost, the infrequent sampling (with an interval of about 7 days) during the rice growing season may have somewhat mischaracterized the seasonal variations in greenhouse gas fluxes and given more weight to individual observations, thereby resulting in biased estimates and potentially increasing the impact of outliers. Therefore, in order to minimize the biased estimates of gas emissions by the chamber-based technique, the time of gas sampling is usually chosen to represent the daily average flux, rather than at times that may lead to overestimation or underestimation of fluxes, as used in previous studies [5,44]. Based on our results, further research is warranted to study real-time monitoring of greenhouse gas emissions by new techniques under different cropping patterns for better estimates of their GWP. In addition, future research should further dissect the mechanisms underlying the changes in the RR cropping pattern in double-season rice regions.

## 4. Materials and Methods

### 4.1. Experimental Site

The field experiment was conducted from October 2020 to October 2021 at the Teaching and Research Comprehensive Base of Hunan Agricultural University in Yanxi Town, Liuyang City, Hunan Province, China (28°30′ N, 113°83′ E). The region belongs to the subtropical monsoon humid climate of the middle and lower reaches of the Yangtze River. The daily meteorological data (minimum and maximum air temperature, and total precipitation) during the experimental period were collected from a nearby weather station, shown in Figure 6. The main cropping pattern in the region is double-season rice. The soil in the experimental site is classified as red-yellow soil, with the following characteristics in the 0–10 cm plow layer: pH, 4.86; bulk density, 1.19 g m^−3^; total organic carbon, 14.77 g kg^−1^; total N, 0.92 g kg^−1^; total phosphorus (P), 1.05 g kg^−1^; total potassium (K), 17.11 g kg^−1^; nitrate, 3.20 mg kg^−1^; ammonium, 25.09 mg kg^−1^; available P, 128.45 mg kg^−1^; and available K, 116.67 mg kg^−1^.

### 4.2. Experimental Design and Agronomic Management

The experiment involved two cropping patterns, fallow-double season rice (DR) and fallow-ratoon rice (RR). In the DR cropping pattern, two varieties of rice were planted for early-season rice and late-season rice, respectively, while two varieties were planted for ratoon rice in the RR cropping pattern. The tested varieties were as follows: Zhongjiazao 17 (ZJZ17) and Liangyou 287 (LY287) were used for early-season rice; Wuyou 103 (WY103) and Taiyou 390 (TY390) were used for late-season rice; and Y Liangyou 911 (YLY911) and Liangyou 6326 (LY6326) were used for ratoon rice. All varieties are widely grown in central China owing to their outstanding performance in the field [18,45,46]. The experiment adopted a split-plot design, with the cropping pattern as the main plot and the varieties as the sub-plot. There were four treatments: ZJZ17 + WY103 (DR1), LY287 + TY390 (DR2), YLY911 (RR1), and LY6326 (RR2), with three replications for each treatment. The main plot was 11.00 m long and 5.60 m wide (with an area of 61.60 m^2^), and embankments with mulching film were built between each main plot to prevent water and fertilizer interflow.

For the DR cropping pattern, the row spacing for both early-season rice and late-season rice was 30.00 cm × 13.30 cm (transplanting density of 25.64 thousand hills ha^−1^, with three seedlings per hill). During the entire growth period of both early-season rice and late-season rice, the fertilization rate was 150 kg N ha^−1^, 90 kg phosphorus pentoxide (P_2_O_5_) ha^−1^, and 120 kg potassium oxide (K_2_O) ha^−1^. For early-season rice, N fertilization was applied as basal, tillering, and panicle fertilizer in a ratio of 2:2:1; phosphorus fertilizer was applied as basal fertilizer in one application; and potassium fertilizer was applied as basal and panicle fertilizer at a ratio of 4:3. After harvest of the early-season rice, the field was prepared and the late-season rice was transplanted. For the late-season rice, the N fertilization was applied as basal, tillering, and panicle fertilizer at a ratio of 4:3:3; phosphorus and potassium fertilizers were applied the same as for early-season rice. Both early-season rice and late-season rice were managed with water management practices of early-stage irrigation, mid-stage field drying, and late-stage alternation of dry and wet conditions. Additionally, disease, insect, and weed control for both early-season rice and late-season rice followed the unified practices implemented for large-scale production in the local region.

For the RR cropping pattern, the row spacing for ratoon rice was the same as that of the DR pattern. The main season fertilization for ratoon rice was 200 kg N ha^−1^, 90 kg P_2_O_5_ ha^−1^, and 120 kg K_2_O ha^−1^. The N fertilization was applied as basal, tillering, and panicle fertilizer at a ratio of 5:2:3. Phosphorus and potassium fertilizers were applied in the same way for early-season rice. For the ratoon season in the ratoon rice, a total of 150 kg N ha^−1^ was applied at a ratio of bud-promoting fertilizer to seedling-promoting fertilizer of 1:1. The bud-promoting fertilizer was applied 10 days after the panicles of the main season had emerged, and the seedling-promoting fertilizer was applied 10 days after the main season harvest. The residual height of the main season for ratoon rice was the height of the plant where the second leaf sheath was bent over. The water management for ratoon rice was as follows: after shallow water transplanting in the main season, deep water was provided to establish good seedlings, followed by field drying. Then, alternation of wet and dry conditions was practiced until the panicle formation stage, after which the water layer in the field was drained. Immediately after harvesting the main season, water was reintroduced, and the water management for the ratoon season followed the moist conditions in the field until the panicle formation stage, after which the water supply was stopped until harvest. Additionally, disease, insect, and weed control for RR followed the same practices as in the DR pattern.

### 4.3. Sample Collection and Analysis

The fluxes of CH_4_, N_2_O, and CO_2_ were determined using a static closed chamber-gas chromatography method [44]. The sampling chamber was made of stainless steel with a diameter of 0.50 m and a height of 0.50 m or 1.10 m (depending on crop height). The top of the chamber had a sampling port connected to a gas sampling three-way valve. A thermometer was installed on the top of the chamber to measure the internal temperature. Additionally, a small fan was placed on the top of the chamber to ensure proper gas mixing inside. Sampling was conducted from 8:30 to 11:00. During the observation, a stainless steel base with grooves (2.50 cm inner diameter and 8.00 cm height) around the outer edge was inserted into the soil to a depth of 5 cm in advance. The sampling chamber was placed on the stainless steel base and sealed by filling the groove with water. The fan was turned on and powered during gas sampling. The sampling time points were 0, 8, 16, and 24 min after the chamber was closed. A 25 mL syringe was used to collect the well-mixed gas from the chamber, which was then transferred into pre-evacuated sealed glass bottles for later analysis in the laboratory. Sampling was conducted in 15-day intervals during the fallow season and 7-day intervals during the rice growing season. The specific sampling dates and frequency were adjusted appropriately based on fertilizer application and precipitation. The concentrations of CH_4_, N_2_O, and CO_2_ were simultaneously analyzed using an Agilent 7890D gas chromatograph. The fluxes were calculated based on the slope of the linear regression equation among the CH_4_, N_2_O, and CO_2_ concentrations of four samples and the sampling time [44]. The cumulative emissions of CH_4_, N_2_O, and CO_2_ were calculated according to Equation (1), as described by Xu et al. [44]. During sampling, the temperature inside the chamber was recorded, and the TDR300 soil moisture meter was used to measure the soil temperature at 7.6 cm soil depth.

(1) $$CE=\sum \left[\frac{\left(F_{i}+F_{i+1}\right)}{2}\times 10^{-3}\times d\times 24\times 10\right]$$

where CE is the cumulative emissions (kg ha^−1^), $F_{i}$ and $F_{i+1}$ are the measured fluxes of two consecutive sampling days (mg m^−2^ h^−1^), and d is the number of days between two adjacent sampling days.

Before rice harvest, investigate the number of effective panicles. Then, select rice with uniform growth in an area of 3 m^2^ per plot for yield measurement. The harvested rice is threshed, and dried, and then impurities are removed through winnowing. The total weight of grains and moisture content are determined. Additionally, the rice yield is calculated based on a moisture content of 14%.

### 4.4. Calculation of GWP and Yield-Scaled GWP

In this study, the GWP was used as a relative indicator to comprehensively assess the potential effects of CH_4_, N_2_O, and CO_2_ emissions on the climate system in the DR and RR cropping patterns. In the GWP estimation, CO_2_ was used as the reference gas, and the changes in CH_4_ and N_2_O emissions were converted into CO_2_ equivalents using GWP. Over a 100-year time scale, the GWP of unit mass of CH_4_ and N_2_O is equivalent to 27.2 times and 273 times that of CO_2_, respectively [47]. The calculation of GWP for greenhouse gas emissions is as follows:

(2) $$GWP\left(kg\;{CO}_{2}-equivalents\;{ha}^{-1}\right)=27.2\times {CE}_{\left({CH}_{4}\right)}+273\times {CE}_{\left(N_{2}O\right)}+1\times {CE}_{\left({CO}_{2}\right)}$$

where ${CE}_{\left({CH}_{4}\right)}$, ${CE}_{\left(N_{2}O\right)}$ and ${CE}_{\left({CO}_{2}\right)}$ are the cumulative emissions of CH_4_, N_2_O and CO_2_ (kg ha^−1^), respectively.

Additionally, while considering the comprehensive greenhouse effect of CH_4_, N_2_O, and CO_2_ emissions under the DR and RR cropping patterns, it is particularly important to comprehensively consider their production benefits. The development of sustainable agriculture requires a harmonious integration of environmental and production benefits. The GWP per unit yield (yield-scaled GWP) in agricultural ecosystems can well reflect the coordination and unity of environment and production benefits, and is suitable for comprehensively evaluating the impact of various agricultural management measures on CH_4_, N_2_O, and CO_2_ emissions from paddy fields. The specific formula for estimation can be expressed as [24]:

(3) $$Yield-scaled\;GWP\left(kg\;{CO}_{2}-equivalents\;per\;kg\;of\;grain\;yield\right)=\frac{GWP}{grain\;yield}$$

### 4.5. Data Analysis

All experimental data were analyzed and organized using Excel (Microsoft Office, 2019). Analysis of variance and multiple comparisons were conducted using the GLM procedure in SAS 9.4 software. The experimental results were presented as the average values and standard errors of three replications. R 4.1.2 software was used for graphical representation.

## 5. Conclusions

Conversion from the DR to RR cropping pattern resulted in changes in greenhouse gas emissions, GWP, and yield-scaled GWP on a short-term scale. Compared with DR, the RR cropping pattern significantly increased the cumulative emissions of CH_4_, N_2_O, and CO_2_ in the first cropping season. Conversely, the RR cropping pattern effectively reduced cumulative emissions of CH_4_, N_2_O, and CO_2_ in the second cropping season. Compared with the DR cropping pattern, the RR cropping pattern showed significantly lower annual cumulative CH_4_ emissions, but no significant difference in cumulative annual N_2_O and CO_2_ emissions. The conversion from the DR cropping pattern to the RR cropping pattern could not only reduce the annual GWP, but also reduce annual yield-scaled GWP. Furthermore, rice variety also showed certain effects on the yields and greenhouse gas emissions in different RR cropping patterns. Compared with the RR1 cropping pattern, the RR2 cropping pattern significantly increased the annual grain yield, while decreasing the annual GWP and annual yield-scaled GWP. These results suggest that the RR2 cropping pattern may be a promising strategy with lower GWP and yield-scaled GWP while ensuring high crop yields in double-season rice regions.

## Figures and Tables

**Table 1:** Analysis of variance in grain yield, greenhouse gases (CH_4_, N_2_O, and CO_2_) mean flux, greenhouse gases cumulative emissions, GWP, and yield-scaled GWP.

Sources	Grain Yield	Mean CH_4_ Flux	Mean N_2_O Flux	Mean CO_2_ Flux	Cumulative CH_4_ Emission	Cumulative N_2_O Emission	Cumulative CO_2_ Emission	GWP	Yield-Scaled GWP
Season (S)	7.69 ** ^1^	4.49 *	31.23 **	0.23 ns	26.39 **	51.80 **	20.18 **	23.69 **	2.57 ns
Cropping pattern (C)	0.17 ns	5.37 *	0.18 ns	0.00 ns	4.72 *	0.07 ns	0.01 ns	0.25 ns	0.29 ns
Variety (V)	0.05 ns	0.00 ns	0.43 ns	0.00 ns	0.00 ns	0.07 ns	0.00 ns	0.00 ns	0.20 ns
S × C	0.47 ns	4.48 *	0.69 ns	0.73 ns	1.61 ns	0.91 ns	0.73 ns	0.94 ns	12.35 **
S × V	0.04 ns	0.00 ns	0.02 ns	0.02 ns	0.03 ns	0.01 ns	0.00 ns	0.00 ns	0.38 ns
C × V	0.47 ns	0.39 ns	1.81 ns	0.05 ns	0.35 ns	0.69 ns	0.07 ns	0.12 ns	6.19 *
S × C × V	0.00 ns	0.01 ns	0.02 ns	0.02 ns	0.00 ns	0.02 ns	0.01 ns	0.01 ns	0.05 ns

^1^ Data are *F* values in the table; ** *p* < 0.01, * 0.01 ≤ *p* < 0.05, ns denotes not significant.

**Table 2:** Dates of sowing, transplanting, and harvest under different cropping patterns in 2021.

Cropping Patterns ^1^	Crop Season	Varieties ^2^	Dates of Sowing-Transplanting-Harvest, mm/dd (Days)
DR	First crop season	ZJZ17	3/23-4/30-7/17 (116)
		LY287	3/23-4/30-7/17 (116)
	Second crop season	WY103	6/25-7/23-10/10 (107)
		TY390	6/25-7/23-10/10 (107)
RR	First crop season	YLY911	3/23-4/30-8/9 (139)
		LY6326	3/23-4/30-8/9 (139)
	Second crop season	YLY911	/-/-10/9 (61)
		LY6326	/-/-10/9 (61)

^1^ DR represents the fallow-double season rice cropping pattern and RR represents the fallow-ratoon rice cropping pattern. ^2^ ZJZ17: Zhongjiazao 17; LY287: Liangyou 287; WY103: Wuyou 103; TY390: Taiyou 390; YLY911: Y Liangyou 911; and LY6326: Liangyou 6326.

## Data Availability

All data generated or analyzed during this study are included in this published article.
